# Precision Medicine in Diabetic Retinopathy: The Role of Genetic and Epigenetic Biomarkers

**DOI:** 10.3390/jcm14248778

**Published:** 2025-12-11

**Authors:** Snježana Kaštelan, Tamara Nikuševa-Martić, Daria Pašalić, Tomislav Matejić, Antonela Gverović Antunica

**Affiliations:** 1 Course Council Fundamentals of Medical Skills, School of Medicine, University of Zagreb, 10000 Zagreb, Croatia; 2 Department of Ophthalmology, Clinical Hospital Dubrava, 10000 Zagreb, Croatia; 3 Department of Biology and Genetics, School of Medicine, University of Zagreb, 10000 Zagreb, Croatia; 4 Department of Medical Chemistry, Biochemistry and Clinical Chemistry, School of Medicine, University of Zagreb, 10000 Zagreb, Croatia; 5 Surgery Clinic, Clinical Hospital Sveti Duh, 10000 Zagreb, Croatia; 6 Department of Ophthalmology, General Hospital Dubrovnik, 20000 Dubrovnik, Croatia

**Keywords:** diabetic retinopathy, genetic biomarkers, epigenetics, precision medicine, genomics, pharmacogenomics, genome-wide association studies (GWAS), personalised medicine, precision ophthalmology

## Abstract

Diabetes mellitus and its microvascular complications, including diabetic retinopathy (DR), present significant health challenges. DR is a leading cause of vision impairment and blindness among working-age individuals in developed countries. The prevalence of DR continues to rise, underscoring the need for more precise diagnostic and therapeutic strategies. Due to its multifactorial nature and despite advancements in understanding DR pathophysiology, predicting its onset and progression remains challenging. Traditional screening and treatment methods often fall short of addressing the heterogeneous nature of the disease, underscoring the need for personalised therapeutic strategies. Recent research has highlighted the vital role of genetic biomarkers in the development and progression of DR, paving the way for a precision medicine approach. Personalised eye care in patients with diabetes aims to accurately predict the risk of DR progression and visual loss in real time. A precision medicine approach that utilises genetic biomarkers offers a promising pathway for personalised diagnosis and treatment strategies. Each DR case is distinct in phenotype, genotype, and therapeutic response, making personalised therapy crucial for optimising outcomes. Advancements in genomics, including genome-wide association studies (GWAS) and next-generation sequencing (NGS), have identified numerous genetic markers associated with DR susceptibility and severity. Emerging evidence underscores the critical role of genetic factors, which account for 25–50% of the risk of developing DR. Advances in identifying genetic markers, such as gene polymorphisms and human leukocyte antigen associations, along with the development of targeted drugs, highlight a promising future for personalised medicine in DR. By identifying specific genetic variants associated with DR, we can enhance prevention and early diagnosis, tailor personalised treatment plans, and more accurately predict disease progression. This represents a critical step toward personalised medicine in DR management. Integrating genetic and epigenetic biomarkers into clinical models may transform DR care through earlier diagnosis and precision-guided interventions, gearing it toward precision ophthalmology.

## 1. Introduction

Diabetic retinopathy (DR), one of the most common and vision-threatening complications of diabetes mellitus (DM), continues to rise in prevalence and remains a major cause of avoidable visual impairment worldwide [1]. It represents the leading cause of vision impairment and blindness among working-age populations worldwide, significantly affecting quality of life and increasing healthcare costs. DM, once seen as a disease of affluent societies, has now become pervasive worldwide, including in developing nations, bringing a correspondingly high burden of DR [2]. The global prevalence of diabetes has been steadily rising, with an estimated 537 million adults affected in 2021, and projections indicate further increases in the coming decades. This number is projected to reach 643 million by 2030 and 783 million by 2045 [3]. DR affects three-quarters of individuals who have had DM for more than 15 years and remains the primary cause of vision loss [4,5]. The rising prevalence of diabetes, driven by sedentary lifestyles, unhealthy diets, and an ageing population, has led to a parallel increase in DR cases, making it a critical public health concern [5,6]. Despite advancements in diabetes management, current screening and treatment strategies remain insufficient in fully addressing the complexity of DR pathogenesis and progression [7,8,9]. This underscores the need for more refined and individualised approaches to improve patient outcomes and reduce the burden of vision loss.

Precision medicine has emerged as a transformative approach in modern healthcare, emphasising tailored prevention, diagnosis, and treatment strategies based on an individual’s genetic, environmental, and lifestyle factors. Moving beyond the traditional “one-size-fits-all” model, precision medicine leverages advanced genetic and molecular insights to customise medical interventions [10]. In diabetes care, this approach holds immense promise for identifying high-risk individuals, predicting disease development, and optimising therapeutic outcomes, particularly in managing complications like DR [11,12,13].

Incorporating precision medicine into diabetes care integrates genetic, molecular, and clinical data to provide personalised interventions. Advances in genomic technologies, such as genome-wide association studies (GWAS) and next-generation sequencing (NGS), have facilitated the identification of genetic markers associated with diabetes and its complications. Using these insights, clinicians can stratify patients based on risk profiles and develop targeted management strategies [8,9,14,15,16]. DR is a multifactorial disease influenced by lifestyle, genetic and environmental factors. While traditional risk factors such as hyperglycaemia, hypertension, and the duration of diabetes play pivotal roles in DR progression, genetic predisposition also significantly contributes to disease susceptibility [8,9,16,17,18,19]. The application of precision medicine in DR management involves leveraging genetic biomarkers to refine screening protocols, predict disease progression, and personalise therapeutic interventions. This tailored approach may enhance early diagnosis, improve treatment efficacy, and ultimately mitigate the risk of vision loss [20,21].

Genetic biomarkers are now recognised as cornerstones of precision medicine, offering valuable insights into disease susceptibility, progression, and treatment response. In the context of DR, numerous genetic variants have been identified that influence key pathological processes, including inflammation, angiogenesis, oxidative stress, and neurodegeneration. Understanding these genetic determinants enables a more comprehensive approach to DR management, allowing for earlier intervention and the development of novel targeted therapies. Emerging evidence suggests that genetic factors contribute 25–50% of the risk for DR development [22,23,24]. Studies have identified specific gene polymorphisms, epigenetic mechanisms, variations in inflammatory and angiogenic pathways, and human leukocyte antigen (HLA) associations that contribute to DR pathogenesis [25,26,27]. By integrating genetic insights into clinical practice, healthcare providers can better predict disease progression, personalise treatment strategies, and enhance patient outcomes. Genetic profiling also facilitates the identification of individuals who may benefit from specific therapeutic interventions, thereby paving the way for more effective and targeted DR management [13,15,28,29,30,31].

This review highlights the substantial public health impact of DR and critically assesses the limitations of current screening and management strategies. In response to the growing need for earlier and more precise risk stratification, this review aims to synthesise current evidence on genetic and epigenetic biomarkers that influence DR susceptibility, progression, and therapeutic response. The primary objective is to delineate how these molecular insights can support the development of precision medicine approaches for personalised prediction, prevention, and management of DR. Additionally, the review identifies key gaps in existing research and outlines future priorities and implementation challenges necessary for translating genetic discoveries into clinical practice.

### Data Collection

This review was conducted as a comprehensive narrative synthesis to integrate current evidence on genetic and epigenetic biomarkers of DR and their relevance for precision medicine. A structured literature search was performed using the PubMed, Scopus, Web of Science, and Google Scholar databases, encompassing all available publications as of July 2025. The search strategy combined the following keywords and Boolean operators: “diabetic retinopathy”, “genetic biomarkers”, “epigenetics”, “genomics”, “GWAS”, “next-generation sequencing”, “inflammation”, “angiogenesis”, “oxidative stress”, “polymorphisms”, “microRNA”, “lncRNA”, “noncoding RNA”, “pathophysiology”, “precision medicine”, “personalised medicine”, “precision ophthalmology”, and “pharmacogenomics”.

The search was restricted to studies published in English. After initial identification, duplicates were removed, and titles and abstracts were screened for relevance. Full-text articles were included if they met the following criteria: addressed genetic, epigenetic, molecular, or clinical biomarkers of DR; investigated their mechanistic, diagnostic, prognostic, or therapeutic significance; or contributed to precision medicine approaches in DR. Studies were excluded if they lacked methodological clarity, did not report primary data, were not aligned with the objectives of this review, or focused on retinal diseases unrelated to DR. Reference lists of all selected studies were manually reviewed (snowballing method) to identify additional relevant publications not captured through the primary search.

Studies meeting the inclusion criteria were qualitatively evaluated, focusing on those published within the last 15 years to ensure inclusion of the most up-to-date molecular and clinical insights into DR pathogenesis. No quantitative meta-analysis was conducted, as the aim of this review was to provide a comprehensive integrative synthesis of the current evidence.

## 2. Pathogenesis of Diabetic Retinopathy

DR results from chronic hyperglycaemia-induced damage to the retinal microvasculature, leading to progressive neurovascular dysfunction. It is a multifactorial disease characterised by progressive microvascular damage in the retina, driven by chronic hyperglycaemia, oxidative stress, inflammation, and neurodegeneration [32,33]. The risk of developing DR correlates with the duration of diabetes, poor glycaemic control, hypertension, and dyslipidaemia [19,34,35]. Clinically, DR progresses through distinct stages, beginning with non-proliferative DR (NPDR), characterised by microaneurysms, intraretinal haemorrhages, and capillary nonperfusion. As the disease progresses, venous beading and cotton-wool spots become more apparent, indicating worsening ischemia. Proliferative DR (PDR) represents the advanced stage, where retinal ischemia triggers the release of pro-angiogenic factors, leading to neovascularisation, vitreous haemorrhage, and vision-threatening complications such as tractional retinal detachment. Diabetic macular oedema (DME) can develop at any stage and results from the breakdown of the blood-retinal barrier, leading to fluid accumulation in the macula and central vision impairment. Some patients exhibit mild or slowly progressing DR. In contrast, others experience rapid deterioration, highlighting the need for predictive biomarkers and personalised treatment strategies [36,37,38].

### 2.1. Molecular Mechanisms

The pathophysiology of DR is complex and involves metabolic dysregulation, oxidative stress, inflammation, vascular dysfunction, retinal neurodegeneration and epigenetic modifications (Table 1) [8,9,17,18,19,20,22,23,24,25,26,27,35,39,40,41,42,43,44,45,46,47,48,49,50,51,52,53,54,55,56,57,58,59]. Chronic hyperglycaemia triggers a cascade of biochemical and molecular changes that contribute to progressive retinal damage.

### 2.2. Neurovascular and Inflammatory Pathways

Persistent inflammation exacerbates vascular dysfunction and neuronal damage. Pericyte loss and capillary degeneration are significant contributors, as pericytes provide structural and functional support to retinal capillaries. Hyperglycaemia-induced pericyte apoptosis leads to microaneurysm formation, capillary dropout, and increased vascular permeability. Additionally, neurodegeneration and glial activation occur early in DR, with ganglion cell apoptosis and Müller cell activation playing key roles in disease progression, emphasising that DR is not solely a vascular disorder but also a neurodegenerative disease [8,9,35,39,40,41,51,55,57,58]. Recent evidence further supports the concept of Functional Diabetic Retinopathy (FDR), which posits that functional impairments occur before clinically detectable vascular lesions are present. Functional DR is characterised by early deficits in retinal function, including altered retinal sensitivity, impaired dark adaptation, and abnormal electrophysiological responses, which reflect neurovascular uncoupling and early neuronal dysfunction preceding structural retinopathy. These changes align closely with the neurodegenerative mechanisms underlying DR pathogenesis, reinforcing the view that neuronal injury is not a secondary phenomenon but an initiating component of DR. Importantly, this new framework highlights the need for functional testing in individuals with diabetes, as FDR represents a preclinical, potentially reversible stage of disease progression, preceding traditional ophthalmoscopic signs of DR [60].

Genetic susceptibility plays a crucial role in DR development, with heritability estimated to be up to 27% for DR and 52% for PDR [30,42,43]. GWAS and candidate gene analyses have identified several genetic loci associated with DR susceptibility. Key genes involved include vascular endothelial growth factor-A (VEGF-A), which regulates angiogenesis and vascular permeability; angiotensin-converting enzyme (ACE), which plays a role in blood pressure regulation and oxidative stress; aldose reductase (AKR1B1), a key enzyme in the polyol pathway implicated in oxidative stress; and HLA polymorphisms, which influence immune response and inflammation [18,22,23,24,25,26,27,44]. Epigenetic modifications, such as DNA methylation and histone modifications, also contribute to DR progression by altering gene expression in response to environmental factors [25,45,46]. While genetic predisposition plays a crucial role, environmental and lifestyle factors significantly modulate disease progression. Poor glycaemic control, systemic hypertension, dyslipidaemia, and smoking are well-established risk factors that exacerbate retinal damage. Socioeconomic status, access to healthcare, and adherence to diabetes management strategies influence DR prevalence and outcomes. Identifying gene-environment interactions will be important in developing personalised interventions to prevent and manage DR effectively [12,19,35,47].

## 3. Current Clinical Biomarkers in Diabetic Retinopathy

Early detection and monitoring of DR are crucial for preventing vision loss; however, current clinical approaches heavily rely on biochemical and imaging biomarkers, which, despite their value, have significant limitations [9,48,49,50,53,55]. Identifying clinical biomarkers has provided critical insights into disease mechanisms and has paved the way for precision medicine strategies in DR management. Among the most widely used biomarkers are biochemical, inflammatory, angiogenic, and imaging markers, which play a pivotal role in clinical practice and research by facilitating the monitoring of disease progression and the assessment of therapeutic responses (Table 2) [7,9,40,41,48,49,50,51,52,53,54,55,56,57,58,59,60,61]. Nevertheless, these widely used biomarkers are predominantly structural and typically become abnormal only after overt microvascular damage. Recent evidence suggests that functional alterations in the retina, including reduced retinal sensitivity, impaired contrast processing, delayed dark adaptation, and abnormal electrophysiological responses, occur significantly earlier than ophthalmoscopically detectable changes. Functional assessments, including microperimetry, contrast sensitivity testing, dark adaptometry, chromatic sensitivity evaluation, photostress recovery testing, and multifocal electroretinography (mfERG), have demonstrated the capacity to detect early neurofunctional deficits before structural abnormalities appear. These modalities support the emerging concept of FDR, which posits that functional decline constitutes the earliest stage of DR pathophysiology. Integrating functional biomarkers alongside traditional structural measures may thus substantially enhance early detection, risk stratification, and timely intervention [60].

### 3.1. Biochemical Biomarkers

Biochemical biomarkers provide insights into the metabolic and vascular dysregulation underlying DR. Chronic hyperglycaemia, oxidative stress, and dyslipidaemia contribute to retinal damage and serve as key indicators of disease progression. One of the most widely recognised biomarkers is haemoglobin A1c (HbA1c), which reflects long-term glycaemic control and correlates with the risk of DR onset and severity [52,53]. Increased serum levels of advanced glycation end products (AGEs) and upregulation of their receptor (RAGE) expression in retinal tissue have been associated with DR progression [54,55].

Dyslipidaemia is another metabolic abnormality implicated in DR pathogenesis. Elevated levels of low-density lipoprotein (LDL) and small dense LDL (sdLDL) are linked to retinal microvascular damage and increased vascular permeability [55]. Oxidative stress markers, including malondialdehyde (MDA) and reduced antioxidant defences such as superoxide dismutase and glutathione, further contribute to endothelial dysfunction in DR [7,53].

### 3.2. Inflammatory Biomarkers

Inflammation is a key driver of DR progression, and numerous inflammatory mediators have been identified as potential biomarkers. Proinflammatory cytokines such as interleukin-1 beta IL-1β, IL-6, IL-8, IL-10, IL-17, tumour necrosis factor-alpha (TNF-α), and monocyte chemotactic protein-1 (MCP-1) are elevated in both systemic circulation and intraocular fluids of DR patients [40,55]. These cytokines contribute to blood-retinal barrier breakdown, endothelial cell activation, and leukocyte infiltration, exacerbating retinal damage [54]. The role of inflammation in DR pathogenesis is further supported by evidence linking endothelial dysfunction and chronic low-grade inflammation to disease severity [57,58].

Adhesion molecules, including intercellular adhesion molecule-1 (ICAM-1) and vascular cell adhesion molecule-1 (VCAM-1), are upregulated in DR and facilitate leukocyte adhesion to the retinal endothelium, further promoting vascular inflammation [7,53]. Pentraxin 3 (PTX3), an acute-phase inflammatory marker, has been identified as a potential indicator of DR severity, with increased levels correlating with more advanced stages [54,55]. Additionally, recent studies suggest that retinol-binding protein 3 (RBP3) may have a protective role in DR, as its levels in aqueous humour decline with disease progression [53]. Inflammatory dysregulation has also been proposed as a key link between metabolic disturbances, obesity, and DR progression [51,58,59].

### 3.3. Angiogenic Biomarkers

Pathological angiogenesis is a hallmark of PDR. VEGF is the principal driver of neovascularisation in DR, with elevated levels in serum, aqueous humour, and vitreous fluid correlating with disease severity. As such, it presents a key target for anti-VEGF therapies [53,54,61]. Other angiogenic biomarkers have also been implicated in DR progression. Placental growth factor (PlGF), a member of the VEGF family, has been shown to contribute to retinal neovascularisation and may serve as a predictive biomarker for disease progression [55]. Angiopoietins (ANG-1 and ANG-2) play a significant role in vascular stability and permeability, with elevated ANG-2 levels disrupting the ANG-1/ANG-2 balance and exacerbating retinal pathology [53,62]. Additionally, fibroblast growth factor (FGF) has emerged as a potential marker of retinal vascular remodelling and inflammation in DR [53,55].

### 3.4. Imaging Biomarkers

Advanced retinal imaging techniques have transformed DR diagnosis and monitoring, enabling high-resolution visualisation of retinal microvascular and neurodegenerative changes. Optical Coherence Tomography (OCT) allows for non-invasive, cross-sectional imaging of retinal layers, providing critical insights into early neuroretinal alterations and DME. OCT angiography (OCTA) further enables detailed assessment of capillary dropout and retinal ischemia without the need for contrast dye, making it a promising tool for early disease detection and progression monitoring [63]. Fluorescein angiography remains a gold standard for evaluating vascular leakage and retinal ischemia, while fundus autofluorescence provides metabolic insights into oxidative stress and lipofuscin accumulation in DR [39,64,65,66]. Automated retinal vessel analysis from fundus photographs enables quantification of changes in vessel calibre, which have been proposed as early indicators of microvascular dysfunction. Furthermore, it could enhance eye care in DM patients by providing more precise DR classification and facilitating widespread screening [67,68].

Despite the advancements in imaging techniques, several limitations persist. Current imaging modalities primarily detect structural changes at relatively advanced disease stages, reducing their effectiveness for early intervention. Additionally, interobserver variability in manual image interpretation and differences in imaging equipment and software algorithms across clinical settings can impact diagnostic consistency [63]. Recent advancements in artificial intelligence (AI) and deep learning have significantly enhanced the screening, detection, and risk assessment of DR. Deep learning algorithms trained on colour fundus photographs, ultra-widefield retinal images, and OCTA scans can accurately predict DR incidence, progression, and the development of vision-threatening complications. These models facilitate early detection, enabling timely intervention and personalised patient management. The integration of AI-driven predictive analytics into clinical workflows has the potential to optimise screening intervals and improve risk stratification, marking a shift toward a precision medicine approach in DR care [67,68,69].

The limitations of current diagnostic and prognostic tools in DR underscore the need for a more comprehensive biomarker-driven approach. Traditional screening programmes, primarily based on fundus photography and clinical risk factors, often fail to capture the heterogeneous nature of DR progression. Moreover, reliance on HbA1c levels and disease duration as primary risk indicators does not fully account for inter-individual differences in metabolic control, inflammatory responses, and microvascular resilience [9,35,38,68,70]. There is a growing demand for a precision medicine framework encompassing diverse biomarker profiles to refine risk prediction and treatment response assessment.

Recent research has explored the value of multi-omics approaches, integrating proteomics, metabolomics, and lipidomics to uncover novel biomarker signatures for DR. Proteomic analyses have identified alterations in retinal and systemic protein expression patterns associated with inflammation, angiogenesis, and neurodegeneration. Metabolomic profiling has revealed distinct metabolic signatures associated with lipid dysregulation and mitochondrial dysfunction in DR patients, offering potential for biomarker discovery. Lipidomic studies have demonstrated that altered lipid metabolism, including dysregulated sphingolipid and ceramide pathways, plays a role in retinal vascular pathology. These omics-based insights, when integrated with traditional clinical markers, could transform early detection strategies and enable more targeted therapeutic interventions [52,56,71,72]. Although clinical and biochemical biomarkers have advanced our understanding of DR, they often fail to explain inter-individual variability in disease progression. Genetic biomarkers, by contrast, capture inherited susceptibility and are central to precision medicine approaches.

## 4. Genetic Biomarkers of Diabetic Retinopathy

While hyperglycaemia-induced vascular damage plays a central role in the pathogenesis of DR, growing evidence suggests that genetic factors significantly influence individual susceptibility and disease progression. Despite substantial progress in understanding the genetic underpinnings of DR, a gap remains in translating these findings into practical clinical applications. Notably, limited efforts have been made to integrate genetic markers into diagnostic algorithms or personalised treatment strategies.

Multiple genetic variants have been implicated in DR pathogenesis, particularly through three key interconnected pathological pathways: angiogenesis, inflammation, and oxidative stress. These mechanisms collectively contribute to retinal damage and neovascularisation, forming the molecular basis of DR and presenting potential targets for early diagnosis and therapeutic intervention.

Pei et al. identified several gene variants associated with increased disease risk, offering new perspectives for early molecular stratification and individualised treatment strategies [12].

### 4.1. Angiogenesis Pathway

Angiogenesis, the formation of new blood vessels, is a hallmark of PDR. Genetic polymorphisms in the VEGF gene, which encodes VEGF, are strongly associated with increased retinal neovascularisation and vascular permeability, contributing to DR progression [12,73]. Variants in VEGF-C have similarly been linked to an elevated risk of DME, further underlining its role in pathological angiogenesis and retinal fluid imbalance [74].

A single-nucleotide polymorphism (SNP) near the GRB2 gene (rs9896052) has been associated with sight-threatening DR, suggesting a role in downstream signalling that modulates neovascular growth [75]. Moreover, Erythropoietin (EPO), known for its hematopoietic function, is upregulated under retinal hypoxic conditions and may contribute to neovascular complications in DR [76].

### 4.2. Inflammation Pathway

Chronic inflammation is a significant driver of DR progression. Elevated levels of inflammatory cytokines such as IL-6 and TNF-α have been consistently observed in DR patients, facilitating vascular leakage, cell apoptosis, and abnormal angiogenesis [77]. Inflammatory mediators also interact with adhesion molecules, such as ICAM-1, which has been correlated with DR severity in meta-analyses [20,78].

### 4.3. Oxidative Stress Pathway

Oxidative stress arises from an imbalance between reactive oxygen species (ROS) and antioxidant defences, leading to retinal tissue damage. Genetic variants in antioxidant enzymes such as SOD1 and SOD2 can affect susceptibility to oxidative injury and thus influence DR onset and severity [79]. The AKR1B1 gene, encoding aldose reductase—a key enzyme in the polyol pathway—has been linked to DR due to its role in ROS production and inflammation [80].

Some genetic variants that contribute to the pathogenesis of DR are presented in Table 3.

### 4.4. Emerging Biomarkers from Omics Technologies

Recent advances in transcriptomics and proteomics have enabled the identification of novel genetic biomarkers that provide insight into the pathophysiology of DR and may inform precision medicine. Cappellani et al. [20] used the OmicsPred PheWAS platform to identify 49 genes and 22 proteins associated with DR and/or DM, further categorising them into condition-specific and overlapping targets. Among the key biomarkers, *AGER* (Receptor for Advanced Glycation End Products) stands out for its involvement in oxidative stress, inflammation, and vascular alterations that contribute to the development of DR. Additionally, the complement system components *C2* and *C4A* point to the activation of immune-mediated damage in the pathogenesis of this disease [20]. These insights emphasise the multifactorial nature of DR and the importance of integrating genetic information with clinical and biochemical data. While reproducibility and effect size remain challenges, these findings pave the way for the development of genetic risk scores and personalised therapies tailored to each patient’s genetic profile.

### 4.5. Critical Appraisal of Previous Research

Despite significant advances in elucidating the genetic underpinnings of DR, existing studies have produced inconsistent and sometimes contradictory findings. For instance, polymorphisms in genes such as VEGF and AKR1B1 have been shown to have strong associations with DR in certain populations. In contrast, the same variants failed to replicate in others, highlighting the influence of ethnic-specific genetic backgrounds and environmental interactions [30,81].

Furthermore, many studies are limited by small sample sizes, insufficient statistical power, and inconsistent phenotypic definitions of DR [82]. The heterogeneity in defining clinical stages—particularly the inconsistent distinction between non-proliferative and proliferative DR—contributes to conflicting results and hinders the identification of robust and reproducible genetic markers [24].

These challenges underscore the urgent need for larger, well-powered longitudinal studies with standardised phenotyping protocols. Uniform diagnostic criteria and consistent classification of DR stages would improve the comparability of results across studies and enhance the discovery of reliable genetic biomarkers, ultimately supporting the development of predictive and personalised approaches to DR management [12]. Beyond protein-coding genes, non-coding RNAs and epigenetic modifiers play a fundamental regulatory role in DR pathogenesis. These molecules modulate gene expression without altering DNA sequences, providing an additional layer of complexity and potential therapeutic targets.

## 5. Non-Coding RNA Molecules and Epigenetic Modifiers in the Pathogenesis of Diabetic Retinopathy

### 5.1. MicroRNA in the Regulation of Diabetic Retinopathy

By binding specific sites in the 3′-untranslated region (3′-UTR) of target mRNAs, microRNA (miRNA), a small non-coding RNA, controls a large number of human protein-coding genes by either preventing translation or promoting mRNA degradation. Therefore, miRNAs have been linked to significant cellular functions in mammals, like haematopoiesis, differentiation, cell-cycle regulation, cell growth, development, and apoptosis [83]. Ocular diseases are among the numerous illnesses associated with aberrant miRNA expression.

Numerous investigations across various human populations have demonstrated the significant role of miR-126 in DR. In a Chinese study conducted in Guangdong Province, patients with diabetes with varying degrees of retinal degeneration (NPDR or PDR) showed significantly reduced miR-126 expression. Furthermore, serum miR-126 levels were found to distinguish patients with DR from healthy controls [84]. Serum miRNA-126 levels were much lower in Egyptian patients with serious diabetes-related problems and those with DR compared to diabetic patients who did not have any noticeable complications [85]. EURODIAB Prospective Complications Study was conducted on a large cohort in cooperation with scientists from Italy, the Netherlands and the United Kingdom. This study also demonstrated that miR-126 has significantly lower expression in diabetic patients with various retinopathy complications in comparison to the control group (without complications) [86].

Endothelial cells are rich in miR-126 and play a crucial role in maintaining endothelial homeostasis and controlling endothelial inflammation. Therefore, downregulation of miR-126 expression may lead to diabetic complications, especially regarding micro-/macrovascular diseases in diabetes [87]. An in vitro diagnostic study, performed on cell cultures of human retinal pericytes (HRP), confirmed downregulation of miR-126 in diabetic-like conditions, as well as approved involvement of extracellular vesicles derived from mesenchymal stem cells in vessel destabilisation [88].

DR progression increased with the increase in miR-151a-5p in Chinese DM patients recruited from the General Hospital of Southern Theatre Command, Guangzhou [89]. This miRNA appears to be involved in DKK3 and MAPK signalling pathways.

Extracellular vesicles (EV) from subjects with DM were used as a sample for the determination of specific miRs in the progression of DR. miR-150-5p, miR-21-3p, and miR-30b-5p analysis showed that these three miRs could be involved in abnormal angiogenesis and therefore serve as prognostic biomarkers for DR [90].

When the serum levels of DR patients were compared to those of healthy controls, miR-139-5p expression was noticeably lower in the former group [89]. In the same study, ARPE-19 cells were treated with both normal and elevated glucose concentrations, and the results showed a significant reduction in miR-139-5p expression at elevated glucose concentrations. Additionally, the same cells exhibited decreased antioxidant properties when treated with a high-glucose concentration solution, whereas those treated with miR-139-5p showed increased antioxidant levels [91].

**Figure 1:**
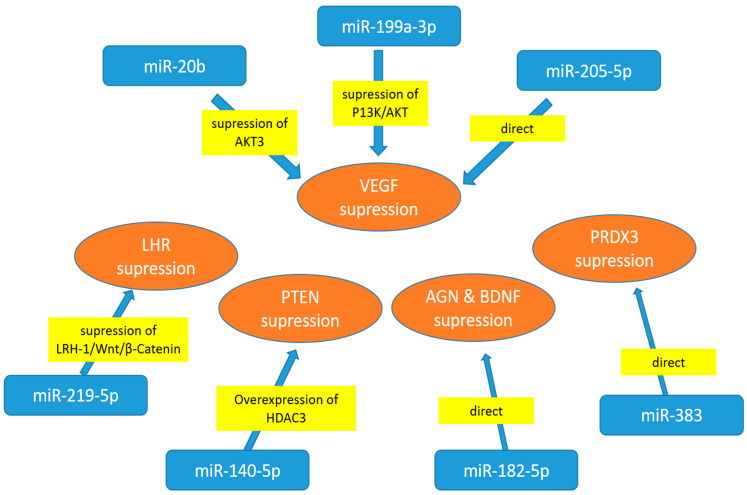
Cell culture experiments with cells exposed to high-glucose treatment—miRNA regulators of specific target proteins. AGN—angiogenin; AKT3-AKT Ser/Thr—protein kinase 3; BDNF—derived neurotrophic factor; LHR-1—liver homologue receptor-1; HDAC3—histone deacetylase 3; PRDX3-thioredoxin-dependent peroxide reductase 3; PTEN—phosphatase and tensin homologue; VEGF—vascular endothelial growth factor.

Many studies have investigated the potential role and therapeutic miR targets in human cell lines, such as ARPE-19, human retinal endothelial cells (HRECs) and human retinal microvascular endothelial cells (HRMECs) (Figure 1). miR-20b, transfected into high-glucose (HG)-treated HRECs, appears to decrease VEGF expression by targeting AKT Ser/Thr—protein kinase 3 (AKT3), the enzyme that regulates cell biosignalling in response to insulin and growth factors. Consequently, miR-20b seems to regulate VEGF-induced events in HRECs under hyperglycaemia [92]. After the treatment of human retinal pigment epithelial (RPE) cell line (ARPE-19) with high-concentration glucose (HG) solution, the expression of miR-219-5p was increased [93]. The same study also demonstrated that liver homologue receptor-1 (LHR-α) is a direct target of miR-219-5p, as HG treatment resulted in significant downregulation of the receptor protein. Another experiment conducted on APRE-19 cells and HRMECs demonstrated that miR-199a-3p suppresses high-glucose-induced angiogenesis by modulating the P13K/AKT pathway and inhibiting VEGF [94]. VEGF-related angiogenesis, which regulates cell signalling pathways (migration, proliferation and apoptosis), can be suppressed by upregulating miR-205-5p. This suggests that miR-205-5p may serve as a therapeutic target for eye proliferative disorders [95].

High glucose concentrations also downregulate miR-140-5p, a microRNA involved in the PI3K/AKT pathway that affects angiogenesis. The study demonstrated that apigenin treatment inhibits proliferation and angiogenesis, suggesting that miR-140-5p may serve as a therapeutic target for DR [96]. The experiment investigated the role of miR-182-5p in regulating the expression of two key angiogenesis factors: angiogenin (AGN) and brain-derived neurotrophic factor (BDNF), using human retinal microvascular endothelial cells (HRECs). Since miR-182-5p overexpression lowers both ANG and BDNF, the study demonstrated that it may be a promising therapeutic target for the treatment of DR [97].

In both ARPE-19 cells and diabetic mice, high hyperglycaemia dramatically increased VEGFA expression while decreasing miR-205-5p levels. These findings suggest that miR-205-5p may be used as a therapeutic approach to treat VEGFA in vascular illnesses associated with proliferative eye disease [98]. In ARPE-19 cells, miR-383 suppressed peroxiredoxin 3 (PRDX3) expression. By suppressing PRDX3 expression, miR-383 overexpression contributes to oxidative stress and cell death in RPE cells. DR may benefit from therapeutic targeting of miR-383 [99].

A study on HRMECSs revealed that diabetic retinas had higher levels of the circulating RNA circ_0001879 [98]. Circ_0001879 binds miRNA-30-3p and functions as a HERMC modulator. Since miR-30-3p was modulated by silencing circ_0001879, the migration of HRMEC and proliferation under high-glucose conditions were suppressed. This suggests that miR-30-3p may be a promising therapeutic target for the treatment of DR [100].

Several investigations in animal models have identified possible pathways of miR involvement in diabetic retinopathy. miR-29b is localised in retinal ganglion cells and the inner nuclear layer of the retina from normal and diabetic rats. It is involved in the regulation of apoptosis through the pro-apoptotic RNA-dependent protein kinase (PKR) signalling pathway. It plays a protective role against apoptosis in both retinal ganglion cells and cells in the inner nuclear layers of the retina [101].

miR-200b is localised in neuronal, glial, and vascular elements of the rat retina. In the rat retina, miR-200b was downregulated, while its target VEGF mRNA and protein were upregulated. Transfection of endothelial cells and intravitreal injection of miR-200b mimic prevented diabetes-induced increases in VEGF mRNA and protein, as well as glucose-induced increases in permeability and angiogenesis [102].

An animal model, performed on streptozotocin-induced diabetic rats, demonstrated differences in the expression of 86 and 120 miRNAs in retinas and retinal endothelial cells, respectively [103]. This study identified an increase in NF-B-, VEGF-, and p53-responsive miRNAs as an important miRNA profile indicative of the continuous pathologic changes in early DR. By inhibiting nuclear factor-kappa B (NF-κB) activation, miR-146 was shown to be a promising therapeutic target for the treatment of DR [103]. Another study of streptozotocin-induced diabetic rats showed increased expression of miR-1273g-3p, which promotes the progression of DR through modulating the autophagy-lysosome pathway [104]. In the mouse model, miR-130a-3p was found to regulate cell division cycle 42 (CDC42). Consequently, reduced miR-130a-3p expression results in elevated CDC42 levels and the progression of DR [105].

### 5.2. Long-Chain Non-Coding RNA (lncRNA) in the Regulation of Diabetic Retinopathy

**Figure 2:**
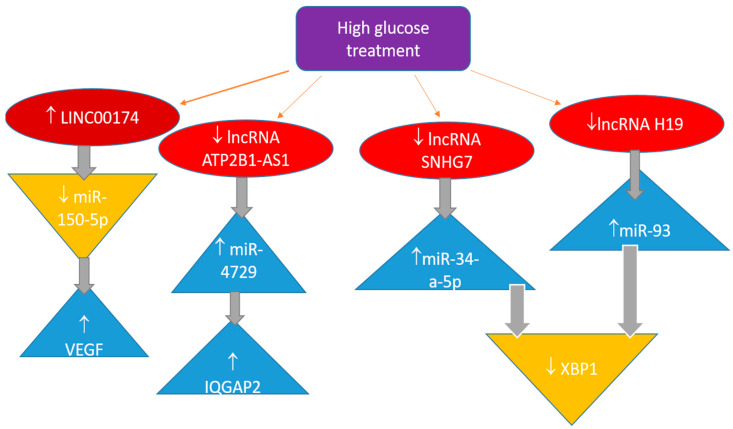
Long non-coding RNA in the regulation of pathobiochemical pathways involved in diabetic retinopathy.

Long non-coding RNAs (lncRNAs) have been well established to influence the progression of DR through various mechanisms. It has been observed that this type of non-coding nucleic acid has a substantial impact on the control mechanisms that regulate gene expression. Mostly, they serve as an additional control step that influences miRNA function (Figure 2).

One of the long intergenic non-coding RNAs is LINC00174. Both DR patients and HRMECs exposed to elevated glucose levels have noticeably greater quantities of this lncRNA [106]. miR-150-5p directly targets VEGFA and binds to the 3′ untranslated region to control VEGFA expression. According to the study’s findings, linc00174 worsens diabetic retinal microangiopathy by controlling the miR-150-5p/VEGFA pathway. These findings suggest an additional therapeutic target for treating DR.

In a study of DR patients and HRECs treated with high hyperglycaemia, miRNA-4729 was elevated while lncRNA ATP2B1-AS1 was downregulated [107]. One of the proteins targeted by miRNA-4729 is IQGAP2, which plays a role in several physiological functions. High-glucose-induced cell proliferation, migration, angiogenesis, and permeability are inhibited by lncRNA ATP2B1-AS1, but miRNA-4729 increases these activities by binding to IQGAP2. Therefore, it appears that the lncRNA ATP2B1-AS1 regulates the miR-4729-IQGAP2 axis to alter endothelial permeability in DR.

Some additional lncRNAs were also investigated, and their effects have been presented in the literature [108,109]. Most lncRNAs act as sponges for specific miRNAs, thereby neutralising the impact of miRNA regulators on particular gene expression.

The role of lncRNA SNHG7 is to act as a sponge, negatively regulating miR-34a-5p. In turn, miR-34a-5p negatively regulates X-box binding protein 1 (XBP1), which is a transcription factor for proteins that play a crucial role in the immune system and the cellular stress response [108]. High glucose levels in cell cultures increase miR-34a-5p production. As a result, increasing lncRNA SNHG7 levels or administering it could yield better outcomes. Consequently, these two non-coding RNAs may serve as potential therapeutic targets. The same target, XBP1, and inflammation in retinal epithelial cells may be influenced by decreased expression of lncRNA H19, which is expected to function as a sponge for miR-93, since the latter miRNA downregulates XBP1 [109]. This interaction suggests a complex regulatory network where lncRNA H19 plays a crucial role in modulating the inflammatory response in retinal epithelial cells. Understanding this relationship could provide insights into novel therapeutic strategies for conditions associated with inflammation and XBP1dysregulation.

As shown, various non-coding RNAs that regulate gene expression may be involved in pathobiochemistry. These non-coding RNAs, including microRNAs and long non-coding RNAs, play crucial roles in modulating different cellular processes, influencing everything from cell differentiation to apoptosis. Understanding their specific functions and mechanisms can provide important insights into disease mechanisms and potential therapeutic targets.

## 6. Genetic–Environmental Interactions in Diabetic Retinopathy

DR arises from a complex interplay between genetic predisposition and environmental exposures. While numerous polymorphisms have been identified in genes regulating angiogenesis, oxidative stress, and inflammation [12,18,20,24,27,30,44,47,75,110], environmental factors, such as obesity, poor metabolic control, hypertension, and smoking, significantly modify their phenotypic expression [8,9,33,34,35,57,58].

*VEGF* and *ACE* gene variants associated with angiogenic activity may produce stronger effects in individuals with inadequate glycaemic control or high oxidative stress [94,98,106,111]. Similarly, obesity-related systemic inflammation amplifies the pathogenic consequences of polymorphisms in cytokine genes such as *IL-6* and *TNF-α*. Cigarette smoking further increases retinal hypoxia and promotes epigenetic modifications, including DNA methylation changes in oxidative stress-related genes [8,9,57,59]. These interactions emphasise that genetic susceptibility does not act in isolation but rather within a broader environmental and metabolic context.

Future research integrating genomic, lifestyle, and metabolic data is therefore essential to define gene–environment interaction networks that determine individual risk trajectories. Such integrative models could refine personalised screening intervals and guide multifactorial interventions targeting both genetic and modifiable risk factors.

## 7. Precision Medicine in Diabetic Retinopathy Management

Conventional screening and treatment strategies for DR often follow a generalised approach, failing to account for individual variations in disease susceptibility, progression, and therapeutic response. Precision medicine seeks to overcome these limitations by integrating genetic, molecular, and clinical data to tailor interventions to individual patients, optimising outcomes and reducing the burden of vision loss [11,13,14,29,39]. Precision medicine in DR is based on identifying patient-specific risk factors and leveraging advances in genomics, transcriptomics, proteomics, and metabolomics. Recent studies with GWAS and NGS have identified numerous genetic variants associated with DR susceptibility and severity [12,27,46,112]. Precision medicine offers more accurate disease prediction and targeted therapeutic strategies by incorporating genetic and molecular data into clinical decision-making [113,114]. Table 4 summarises the essential components and practical applications of precision medicine in DR management [12,13,18,29,30,31,39,113,114,115,116,117,118,119,120,121,122].

### 7.1. Pharmacogenomics and Personalised Therapy

Pharmacogenomics, a key component of precision medicine, is crucial in tailoring DR treatments. Variability in response to anti-VEGF therapy, a mainstay treatment for PDR and DME, has been linked to genetic polymorphisms in the VEGF gene and other angiogenic factors [115]. Studies indicate that patients with specific VEGF genotypes may experience differential responses to anti-VEGF drugs. This suggests that pharmacogenomic profiling could guide treatment selection and dosing [30,116]. Beyond VEGF, genetic markers in inflammatory and neuroprotective pathways, such as TNF-α, IL-6, and BDNF, have been implicated in DR pathophysiology and therapeutic response [12,117,118,119]. Identifying these markers may enable more personalised treatment approaches, reducing treatment failures and adverse effects.

### 7.2. AI and Multi-Omics Integration

The role of precision medicine extends beyond pharmacogenomics to include personalised screening and monitoring strategies. Current DR screening guidelines rely primarily on periodic fundus photography or OCT at predefined intervals. However, AI-driven predictive models integrate genetic, metabolic, and imaging biomarkers to assess disease progression in real time [39,120]. AI-based deep learning algorithms have demonstrated high accuracy in detecting early DR changes, offering an opportunity for individualised screening intervals based on personalised risk assessments [31,113].

Another promising avenue in precision medicine is the use of multi-omics approaches to identify novel therapeutic targets. Metabolomic and proteomic studies have uncovered biomarkers that distinguish patients with progressive DR from those with stable disease, providing new insights into disease pathophysiology. Lipid metabolism and mitochondrial function alterations have been associated with DR progression, suggesting potential targets for precision therapies. Additionally, epigenetic modifications, such as DNA methylation and histone acetylation, may influence DR susceptibility and serve as novel therapeutic targets [45,121,122].

### 7.3. Ethical and Clinical Implementation Challenges

Despite these advancements, several challenges remain in implementing precision medicine in DR management. DR genetic and molecular heterogeneity necessitates large-scale multi-ethnic studies to validate predictive models and treatment algorithms [10,12,14]. Moreover, integrating precision medicine into routine clinical practice requires the development of cost-effective genetic screening tools and policies to ensure equitable access. Ethical considerations, including data privacy and genetic counselling, must also be addressed to promote the responsible use of gene testing in DR care [12,13,18].

**Figure 3:**
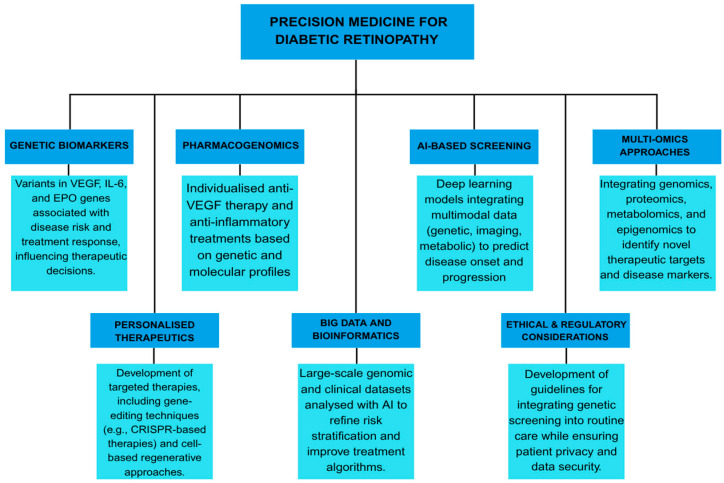
Translational roadmap for implementing genetic and epigenetic biomarkers into precision medicine for diabetic retinopathy. The roadmap outlines the progression from molecular discovery to clinical application, encompassing the identification of genetic biomarkers (e.g., VEGF, IL-6, EPO), pharmacogenomic profiling for individualised therapy, and AI-assisted screening integrating multimodal and multi-omic data. It highlights the development of personalised therapeutic strategies, including gene- and cell-based treatments, supported by continuous feedback between research, clinical validation, and regulatory assessment. Integration of big data analytics and adherence to ethical and regulatory standards ensure the safe and effective translation of precision medicine into diabetic retinopathy care.

Future directions in precision medicine for DR include expanding real-world data registries and clinical trials investigating genotype-guided therapies. Advances in machine learning and bioinformatics will further enhance the integration of multi-omics data, enabling more refined risk stratification and therapeutic personalisation [29,31]. Ultimately, the convergence of precision medicine, AI, and patient-centred care holds great promise for improving DR outcomes and achieving the broader goal of personalised medicine in ophthalmology [31,110,120]. Implementing these principles will require a multidisciplinary consortium that brings together ophthalmologists, diabetologists, geneticists, bioinformaticians, health-economics experts, and patient advocates. It will rely on phased financing from foundations, public grant programmes, and industry partnerships. These steps directly address analytical and clinical validity as well as clinical utility, the three pillars required for clinical adoption. The proposed translational roadmap for integrating validated genetic and multi-omic biomarkers into clinical practice is illustrated in Figure 3.

Despite advances in implementing genetic and multi-omic biomarkers into clinical management of diabetic retinopathy, several scientific and practical barriers continue to limit clinical translation. Future research directions must therefore address validation, standardisation, and ethical considerations to ensure the safe and effective adoption of precision medicine in DR.

## 8. Future Directions and Research Priorities

Despite significant advances in identifying DR-associated genetic biomarkers, translating these findings into routine clinical practice remains challenging. One of the primary barriers is the genetic and molecular heterogeneity of DR, necessitating large-scale, multi-ethnic GWAS and NGS to validate predictive models and identify population-specific risk factors [30]. Moreover, integrating genetic screening into routine ophthalmologic care requires the development of cost-effective testing methods and clear guidelines for clinical implementation, enabling individualised follow-up intervals and timely therapeutic intervention [112].

A promising future direction is to apply multi-omic approaches, integrating genomics, transcriptomics, proteomics, metabolomics, and epigenomics to develop more comprehensive stratification risk models. Recent studies have highlighted the role of metabolomic and lipidomic profiles in distinguishing between progressive and non-progressive DR, opening new avenues for targeted therapy [121,122]. Future research should prioritise large-scale genomic studies, the development of cost-effective genetic tests, and interdisciplinary collaborations to accelerate the translation of genetic discoveries into clinical practice. By leveraging the power of genetic biomarkers, the field is advancing toward a more predictive, preventive, and personalised approach to DR care. Additionally, epigenetic modifications, such as DNA methylation and histone acetylation, may serve as novel therapeutic targets for early intervention [45]. In the context of personalised therapy, pharmacogenomic evidence suggests that *VEGF* and *KDR* variants may influence the response to anti-VEGF agents. At the same time, polymorphisms in *ICAM1* and oxidative stress-related genes may modulate the efficacy of corticosteroids [24,123,124,125]. Future genotype-guided clinical trials should investigate whether specific allelic variants predict therapeutic response, adverse effects, or disease progression, paving the way for biomarker-based treatment algorithms. Ethical, regulatory, and economic considerations, including informed consent, data privacy, and equitable access to genetic testing, must be addressed in parallel to ensure the responsible clinical implementation of genetic testing [120].

A major current gap is the lack of functional validation. Mechanistic studies using in vitro and in vivo models, including CRISPR/Cas9-edited retinal cells, iPSC-derived systems, and transgenic animal models, are crucial to elucidate how specific variants and non-coding RNAs regulate angiogenesis, neurodegeneration, and inflammation in the diabetic retina [126,127].

Successful clinical translation further depends on methodological standardisation and quality assurance. The development of unified operating procedures for sample collection, DNA/RNA extraction, and bioinformatics workflows, together with inter-laboratory validation, will ensure analytical robustness and reproducibility. Pilot implementation studies in tertiary diabetes–retina centres should assess feasibility, cost-effectiveness, and clinical utility, providing data to inform future clinical guidelines on when, how, and for whom genetic testing should be recommended in DR management [120].

AI and machine learning tools have emerged as transformative tools in precision medicine, enabling the integration of genetic and imaging biomarkers to improve the accuracy of DR prediction and treatment optimisation. AI-driven deep learning algorithms have demonstrated high accuracy in predicting DR onset and progression when incorporating genetic risk factors, systemic metabolic data, and multimodal imaging findings [31,120]. Future research should focus on validating these AI models across diverse populations, refining their clinical application, and ensuring seamless integration into ophthalmic care.

From a therapeutic perspective, advances in pharmacogenomics are paving the way for individualised treatment strategies. Genetic polymorphisms in key angiogenic and inflammatory pathways, such as VEGF, IL-6, and erythropoietin, have been shown to influence patient response to anti-VEGF therapy and corticosteroids [24,110,118]. Identifying patient-specific genetic markers may help refine treatment selection, improving therapeutic efficacy and minimising adverse effects. Combining genetic profiling with AI-driven predictive models could revolutionise patient-specific therapeutic approaches.

Ethical and regulatory considerations remain central to the successful implementation of genetic screening in DR management. Issues such as genetic privacy, data security, and the need for genetic counselling must be addressed to ensure the responsible clinical application of genetics. Furthermore, disparities in access to precision medicine technologies must be mitigated through policy-driven initiatives that promote equitable healthcare solutions [13,14].

Finally, successful implementation in clinical practice will require comprehensive health economic and feasibility studies evaluating sustainability, cost-effectiveness, and stakeholder acceptance. International collaboration among ophthalmologists, diabetologists, geneticists, and data scientists is vital to standardise phenotypic definitions, harmonise analytical methodologies, and promote equitable application of genomic medicine across populations. Through these coordinated efforts, genetic and epigenetic biomarkers can evolve from research tools into actionable instruments for precision prevention, diagnosis, and management of DR.

## 9. Conclusions

The rapid advancements in genetic research and precision medicine offer significant promise terms of in managing DR. Genetic biomarkers provide crucial insights into disease susceptibility, progression, and treatment response, enabling the development of personalised therapeutic strategies. However, several challenges remain, including genetic heterogeneity, limited clinical validation, and the incorporation of multi-omics data into routine care. Addressing these challenges requires large-scale collaborative research, technological innovation, and engagement with ethical concerns. Future directions in DR management will likely involve AI-driven predictive models, polygenic risk assessments, and pharmacogenomic interventions, paving the way for personalised ophthalmic care. By leveraging these advancements, precision medicine can transform DR treatment, ultimately improving patient outcomes and reducing the burden of diabetic vision loss. Together, these translational and research efforts outline a feasible roadmap for integrating validated genetic and epigenetic biomarkers into the personalised management and the prevention of DR, aligning with the goals of next-generation precision ophthalmology.

## Figures and Tables

**Table 1:** Key molecular and cellular mechanisms contributing to the pathogenesis of diabetic retinopathy.

Pathogenic Mechanism	Key Molecular Players	Pathophysiological Impact	Clinical Relevance
Metabolic Dysregulation	Polyol pathway (aldose reductase), AGEs, hexosamine pathway	Mitochondrial dysfunction, apoptosis	Contributes to progressive DR severity
Hyperglycaemia-Induced Oxidative Stress	ROS, NADPH oxidase, mitochondrial dysfunction	Endothelial dysfunction, increased permeability	Early vascular damage and microaneurysm formation
Chronic Inflammation	TNF-α, IL-1β, IL-6, IL-8, IL-10, IL-17, NF-κB	Induction of inflammatory cascades, leukostasis, endothelial injury	Progression to DME and neovascularisation
Angiogenesis and Vascular Dysfunction	VEGF-A, angiopoietins, HIF-1α	Increased vascular permeability and neovascularisation	Target for anti-VEGF therapies
Neurodegeneration	BDNF, NMDA receptors, retinal ganglion cells	Retinal neuronal apoptosis and loss of neuroprotection	Associated with early DR progression and visual decline
Epigenetic Modifications	DNA methylation, histone modifications, non-coding RNAs	Gene expression dysregulation	Potential biomarker and therapeutic target

AGEs: Advanced glycation end-products; DR: diabetic retinopathy; ROS: Reactive oxygen species, NADPH: Nicotinamide adenine dinucleotide phosphate; TNF-α: tumour necrosis factor-alpha; IL: interleukin; IL-1β: IL-1 beta, NF-κB: nuclear factor-kappa B; DME: diabetic macular oedema, VEGF: vascular endothelial growth factor, HIF-1α: hypoxia-inducible factor-1α, BDNF: Brain-derived neurotrophic factor, NMDA: N-Methyl-D-aspartate; DNA: Deoxyribonucleic acid; RNA: Ribonucleic acid.

**Table 2:** Principal non-genetic biomarkers of diabetic retinopathy and their clinical relevance.

Biomarker Type	Specific Biomarkers	Biological Role	Clinical Application
Inflammatory Biomarkers	CRP, TNF-α, IL-1β, IL-6, IL-8, IL-10, IL-17	Mediate inflammation and endothelial dysfunction	Predictive marker for DR severity and progression
Angiogenic Factors	VEGF-A, PlGF, Angiopoietins	Promote neovascularisation and vascular leakage	Target for anti-VEGF therapy
Oxidative Stress Biomarkers	MDA, SOD, GPx	Indicate oxidative damage and metabolic stress	Monitor oxidative damage progression
Endothelial Dysfunction Biomarkers	Soluble ICAM-1, VCAM-1, E-selectin	Reflect vascular inflammation, leukocyte adhesion and permeability changes	Early indicators of microvascular dysfunction
Retinal Neurodegeneration Biomarkers	BDNF, GFAP	Associated with retinal ganglion cell loss and glial reactivity	Potential target for neuroprotective therapies
Metabolomic Biomarkers	1,5-anhydroglucitol, lactate, glutamate	Indicate metabolic dysregulation and mitochondrial stress	Emerging marker for early DR detection

TNF-α: tumour necrosis factor-alpha; IL-1β: interleukin-1 beta; CRP: C-reactive protein; DR: diabetic retinopathy; VEGF: vascular endothelial growth factor; PlGF: placental growth factor; MDA: malondialdehyde; SOD: superoxide dismutase; GPx: glutathione peroxidase; ICAM-1: intercellular adhesion molecule-1; VCAM-1: vascular cell adhesion molecule-1; BDNF: brain-derived neurotrophic factor; GFAP: glial fibrillary acidic protein.

**Table 3:** Selected genetic loci and variants implicated in diabetic retinopathy pathogenesis.

Gene(s)/Loci	Function/Association with DR	Reference
SUCNR1 (GPR91)	Activated under hypoxic conditions; promotes angiogenesis	[73]
GLUT1 (SLC2A1)	Involved in glucose transport and retinal metabolic regulation	[8]
ZWINT-MRPS35P3, TCF7L2 (SNPs)	Associated with altered glucose metabolism and retinal cell function	[18]
EYA2, MPDZ, NTNG1, CTAGE14P, MREGP1	Identified in GWAS analyses; potential roles in retinal maintenance	[30]
TBC1D4-COMMD6-UCHL3, LRP2-BBS5, ARL4C-SH3BP4	Identified in Chinese populations with PDR; involved in insulin signalling, apoptosis, and inflammation	[24,44]
CCDC7, ITGB1	Variants associated with vascular integrity and development in type 1 diabetes	[7,77]
STT3B, PALM2, EHD3	Additional loci implicated in DR via GWAS analyses	[12]

SUCNR1 (GPR91): Succinate Receptor 1 (also known as G Protein–Coupled Receptor 91); GLUT1 (SLC2A1): Solute Carrier Family 2 Member 1—Glucose Transporter Type 1; ZWINT: ZW10 Interacting Kinetochore Protein; MRPS35P3: Mitochondrial Ribosomal Protein S35 Pseudogene 3; TCF7L2: Transcription Factor 7 Like 2; EYA2: EYA Transcriptional Coactivator and Phosphatase 2; MPDZ: Multiple PDZ Domain Protein; NTNG1: Netrin G1; CTAGE14P: CTAGE Family Member 14 Pseudogene; MREGP1: Melanoregulin Pseudogene 1; TBC1D4: TBC1 Domain Family Member 4; OMMD6: COMM Domain Containing 6; UCHL3: Ubiquitin C-Terminal Hydrolase L3; LRP2: Low-Density Lipoprotein Receptor–Related Protein 2; BBS5: Bardet–Biedl Syndrome 5; ARL4C: ADP Ribosylation Factor Like GTPase 4C; SH3BP4: SH3 Domain Binding Protein 4; CCDC7: Coiled-Coil Domain Containing 7; ITGB1: Integrin Subunit Beta 1; STT3B: STT3 Oligosaccharyltransferase Complex Catalytic Subunit B; PALM2: Paralemmin 2; EHD3: EH Domain Containing 3.

**Table 4:** Key components and applications of precision medicine in diabetic retinopathy management.

Component	Description	Application in DR
Genetic Biomarkers	Identification of genetic variants influencing DR susceptibility and progression	GWAS, whole-exome sequencing, polygenic risk scores for individualised risk assessment
Pharmacogenomics	Analysis of genetic variations affecting drug metabolism and therapeutic response	Personalised anti-VEGF, corticosteroid, and anti-inflammatory therapy selection
AI-Based Risk Stratification	Machine learning and deep learning models combining genetic, imaging, and clinical data	Development of predictive models for DR onset, progression, and treatment response
Multi-Omics Integration	Integration of genomic, proteomic, metabolomic, and epigenomic data	Refining disease classification, discovering novel biomarkers, and optimising therapy
Targeted Gene Therapies	Gene-editing technologies such as CRISPR-Cas9 for modifying disease-associated pathways	Potential future applications in DR prevention and treatment
Personalised Screening Strategies	Adjusting screening frequency and methods based on individual risk factors	AI-driven risk models for high-risk population identification and monitoring

DR: diabetic retinopathy; AI: artificial intelligence; GWAS: genome-wide association studies.
